# Supplementary material for: A small molecule inhibitor of PTP1B and PTPN2 enhances T cell anti-tumor immunity
Source: Nat Commun. 2023 Jul 27;14:4524. doi: 10.1038/s41467-023-40170-8 (PMC10374545; doi:10.1038/s41467-023-40170-8)
Supplement: Supplementary file 1 — Supplementary Information [file 41467_2023_40170_MOESM1_ESM.pdf]

SUPPLEMENTAL DATA

A small molecule inhibitor of PTP1B and PTPN2 enhances T cell anti-tumor immunity

Shuwei Liang [1, 2 #], Eric Tran [3 #], Xin Du [1, 2], Jiajun Dong [4], Harrison Sudholz [1, 2], Hao Chen [6, 7], Zihan Qu [5], Nicholas D., Huntington [1, 2], Jeffrey J. Babon [6, 7], Nadia J. Kershaw [6, 7], Zhong-Yin Zhang [4,5], Jonathan B. Baell [3, 8], Florian Wiede [1, 2*], and Tony Tiganis [1, 2 *]

[1] Monash Biomedicine Discovery Institute, Monash University, Clayton, Victoria 3800.
[2] Department of Biochemistry and Molecular Biology, Monash University, Clayton, Victoria 3800, Australia.
[3] Monash Institute of Pharmaceutical Sciences, Monash University, Parkville, Victoria 3052, Australia.
[4] Department of Medicinal Chemistry and Molecular Pharmacology, Purdue University, West Lafayette, IN 47907, USA,
[5] Department of Chemistry, Purdue University, West Lafayette, IN 47907, USA,
[6] Walter and Eliza Hall Institute of Medical Research, Parkville, Victoria 3052, Australia.
[7] Department of Medical Biology, The University of Melbourne, Victoria 3052, Australia.
[8] Lyterian Therapeutics, South San Francisco, California 94080, United States of America.

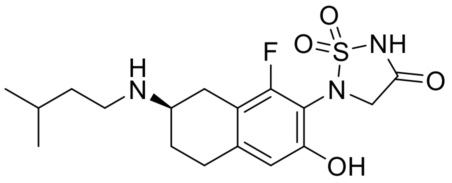

ABBV-CLS-484

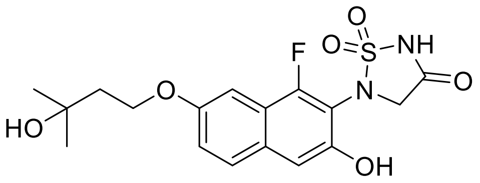

Compound 182

Supplementary Fig. 1. The chemical structures of ABBV-CLS-484 and Compound 182.

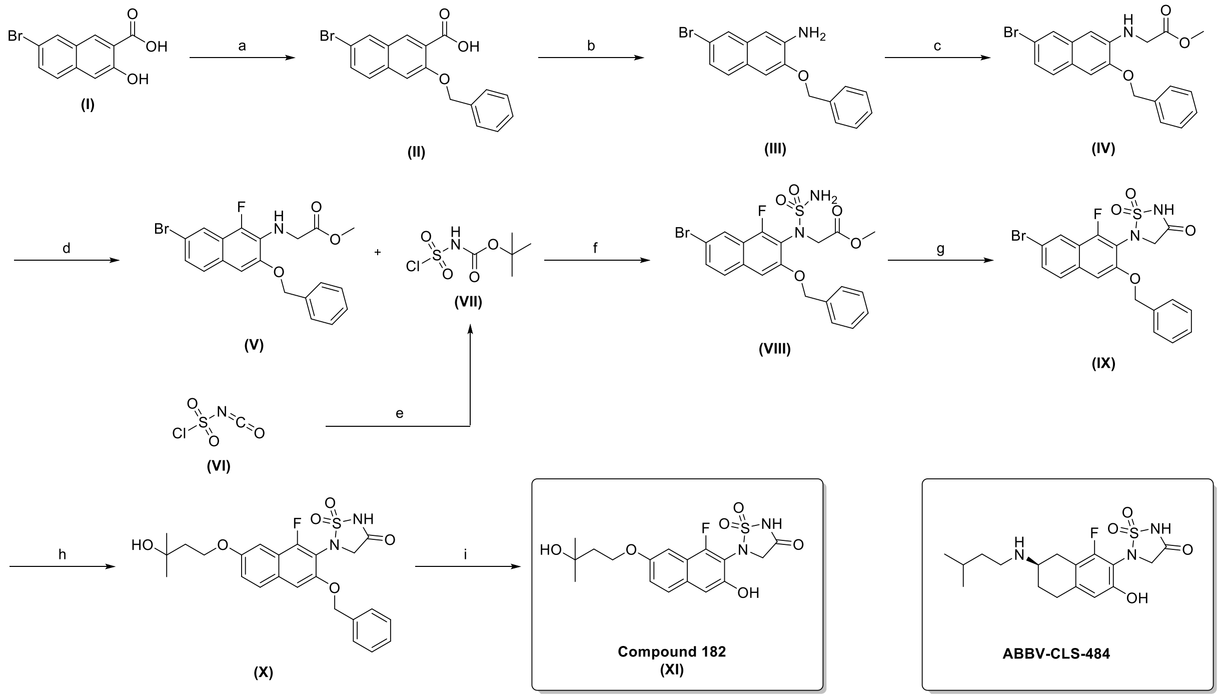

Supplementary Fig. 2. Synthetic Scheme for Compound 182[a] and structure of clinical candidate ABBV-CLS-484. [a]Reagents and conditions: (a) (ii) Benzyl alcohol, Cs_2CO_3, DMF, r.t., 16 h, (ii) LiOH, MeOH/H_2O, 70 °C, 16 h, 94% over 2 steps; (b) (i) diphenylphosphoryl azide, Et_3N, *tert*-butanol / toluene, 80 °C, 16 h, (ii) diethylenetriamine, 130 °C, 16 h, 40% over 2 steps; (c) methyl 2-bromomethylacetate, K_2CO_3, 60 °C, 16 h, 77%; (d) NFSi, THF, r.t., 6 h, 62%; (e) *tert*-butanol, 0 °C, 1 h; (f) (i) Et_3N, 0 °C – r.t, 2 h, (ii) TFA/CH_2Cl_2, r.t., 1 h, 71% over 2 steps; (g) *t*BuOK, THF, r.t., 1 h, 90%; (h) (i) RockPhos Pd G3, Cs_2CO_3, DMF, H_2O, 80 °C, 3 h, (ii) 4-bromo-2-methylbutan-2-ol, Cs_2CO_3, r.t., 16 h, 53%; (i) H_2, Pd/C, MeOH, r.t., 4 h, 64%.

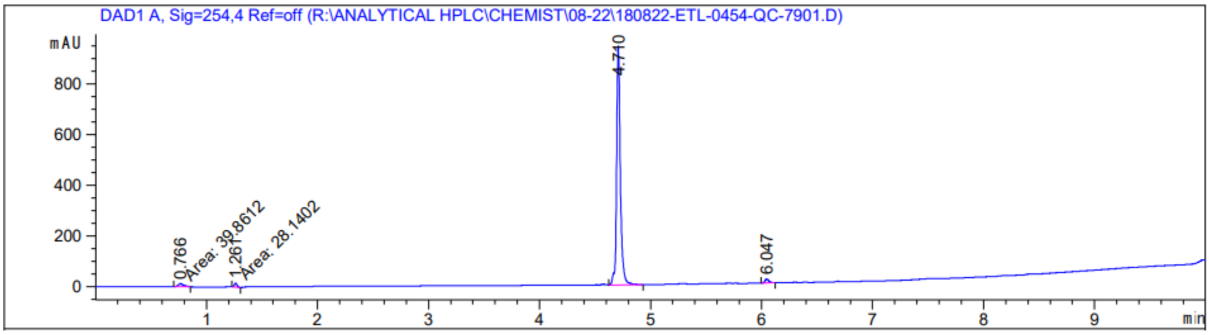

Peak #	RetTime [min]	Type	Width [min]	Area [mAU*s]	Height [mAU]	Area %
1	0.766	MM	0.0549	39.86118	12.11183	1.7379
2	1.261	MM	0.0280	28.14021	16.72783	1.2269
3	4.710	BV	0.0351	2189.17505	942.49902	95.4455
4	6.047	BB	0.0364	36.46122	15.54548	1.5897

Supplementary Fig. 3. HPLC Chromatogram of Compound 182 and Purity. HPLC chromatogram of XI (Compound 182; absorbance measured at wavelength 254 nm) and purity measurement. The compound had a retention time of 4.710 min and a purity of 95% based on peak area (area under the curve).

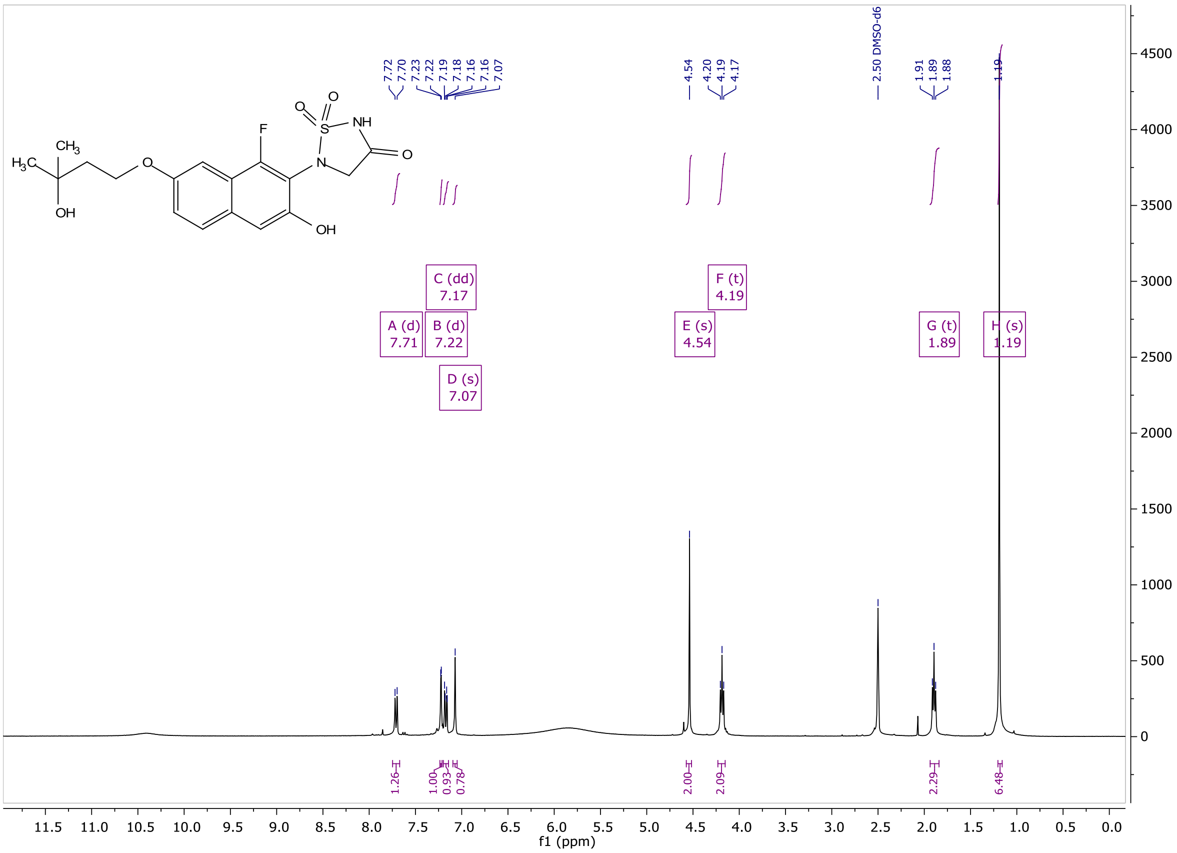

Supplementary Fig. 4. ^{1}H NMR spectrum of Compound 182. ^{1}H NMR spectrum of XI (Compound 182) obtained in deuterated DMSO-d_6 at 400 MHz.

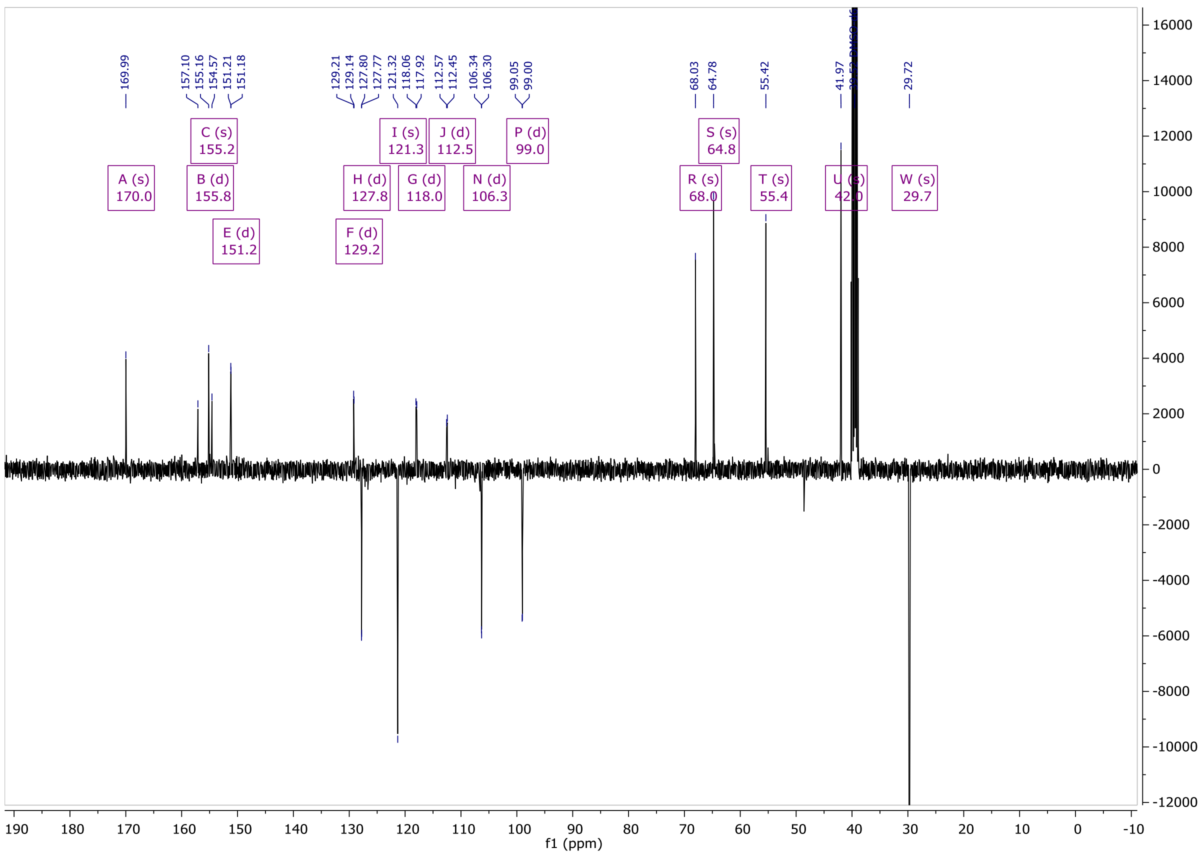

Supplementary Fig. 5. ^{13}C NMR spectrum of Compound 182. ^{13}C-DEPTQ NMR spectrum of XI (Compound 182) obtained in deuterated DMSO-d_6 at 101 MHz.

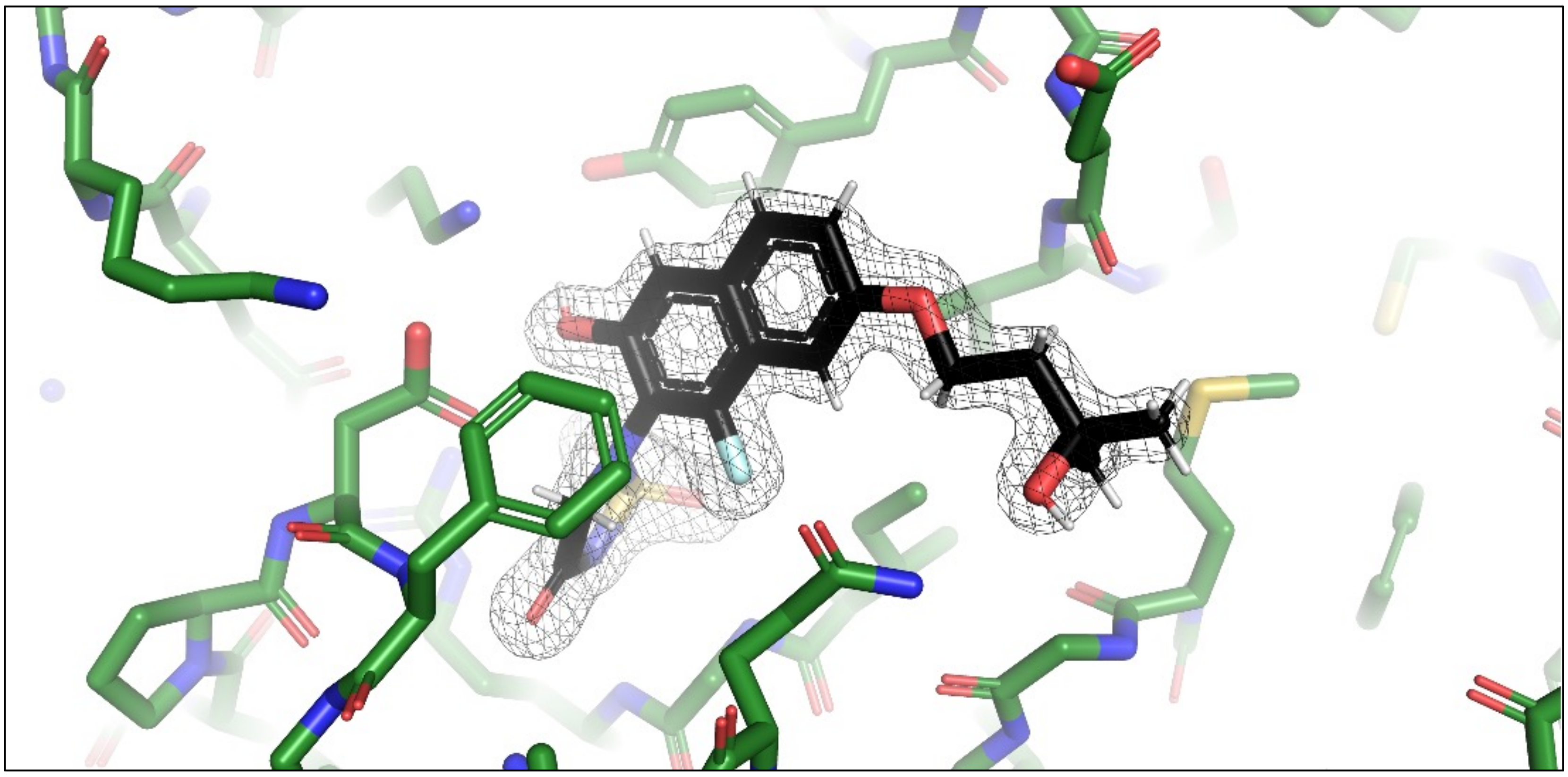

Supplementary Fig. 6. Electron density for Compound 182. The region about the compound in the PTP1B/Compound 182 complex 2Fo-Fc electron density map is shown as a mesh and is contoured at 2σ. See Table 2 for crystallographic details.

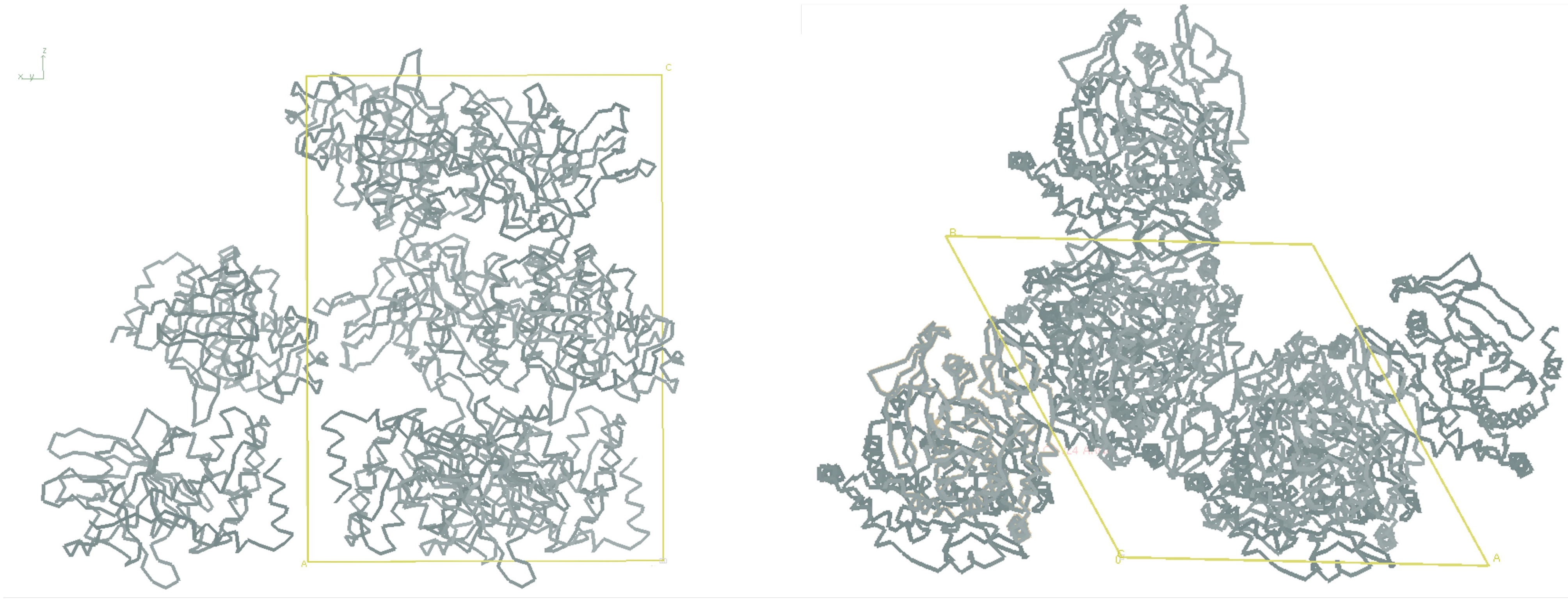

Supplementary Fig. 7. PTP1B crystal structure. Example of the crystal lattice, with the asymmetric unit show in yellow.

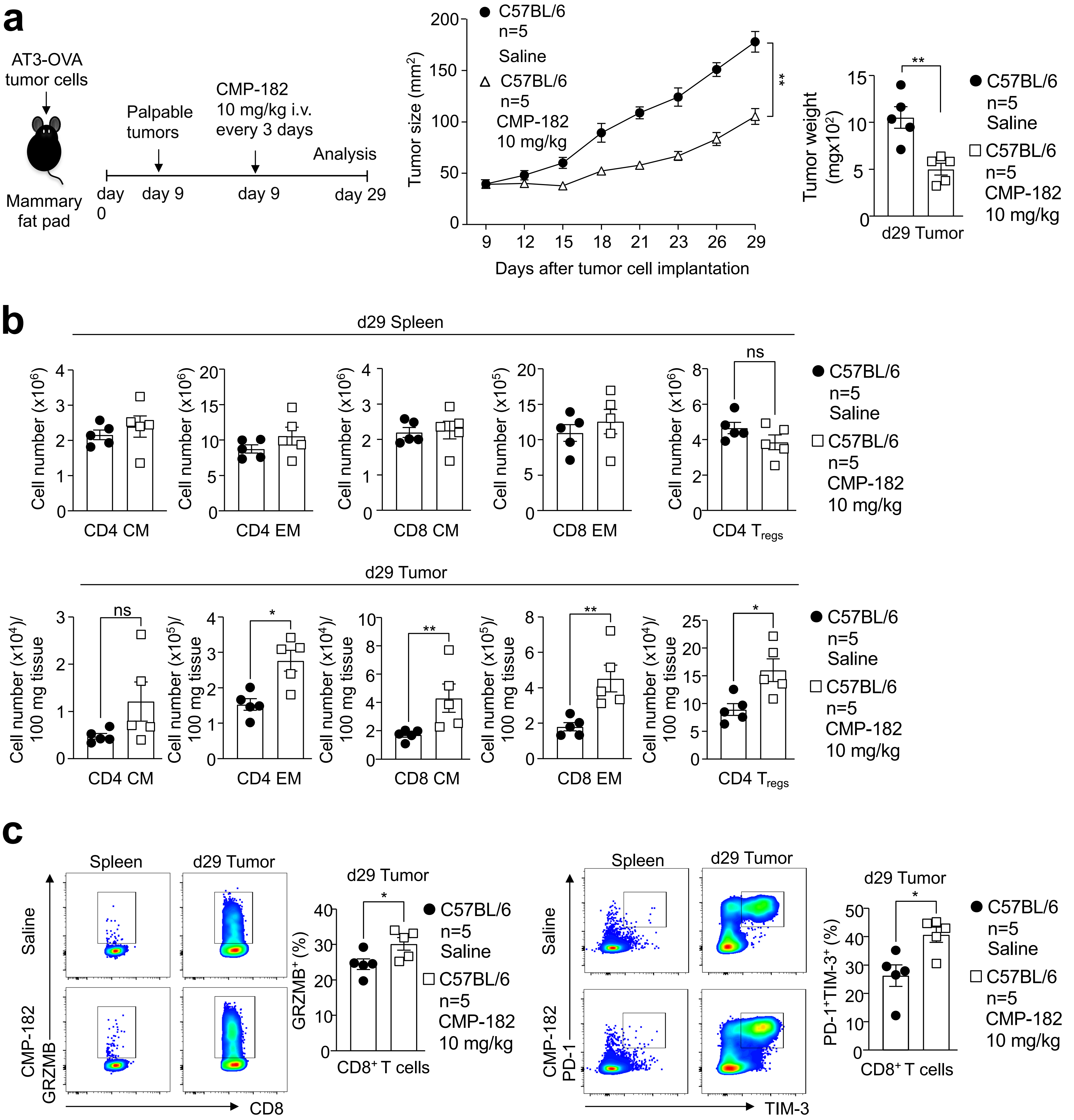
a
AT3-OVA tumor cells
Mammary fat pad
Palpable tumors
CMP-182 10 mg/kg i.v. every 3 days
Analysis
day 0
day 9
day 9
day 29
Tumor size (mm²)
Days after tumor cell implantation
C57BL/6 n=5 Saline
C57BL/6 n=5 CMP-182 10 mg/kg
**
Tumor weight (mgx10²)
d29 Tumor
b
d29 Spleen
Cell number (x10⁶)
Cell number (x10⁵)
CD4 CM
CD4 EM
CD8 CM
CD8 EM
CD4 Tregs
ns
d29 Tumor
Cell number (x10⁴)/ 100 mg tissue
Cell number (x10⁵)/ 100 mg tissue
*
c
Spleen
d29 Tumor
Saline
CMP-182
GRZMB
CD8
GRZMB⁺ (%)
CD8⁺ T cells
PD-1
TIM-3
PD-1⁺TIM-3⁺ (%)

Supplementary Fig. 8. Administration of Compound 182 every three days represses tumor growth. AT3-OVA mammary tumor cells were injected into the fourth inguinal mammary fat pads of 8 week-old C57BL/6 female mice. Mice were treated with Compound 182 (CMP-182; 10 mg/kg i.v.; n=5) or saline (n=5) on days (d) 9, 12, 15, 18, 21, 24 and 27 after tumor cell implantation. **a**) Tumor growth was monitored and tumor weights measured. **b**) Tumor-infiltrating lymphocytes or splenocytes including $CD44^{hi}CD62L^{hi}$ $CD8^+$ and $CD4^+$ central/memory (CM) T cells and $CD44^{hi}CD62L^{lo}$ $CD8^+$ and $CD4^+$ effector/memory (EM) T cells and $CD4^+CD25^+FoxP3^+$ regulatory T cells (T_{regs}) were analysed by flow cytometry. **c**) Tumor-infiltrating T cells from (a) were assessed for intracellular granzyme B (GRZMB) and surface PD-1 and TIM-3 in unstimulated tumor-infiltrating $CD8^+$ T cells. In (a-c) representative results (means ± SEM) from at least two independent experiments are shown. Significance for tumor sizes in (a) was determined using a 2-way ANOVA Test and for tumor weights in (a) using a 2-tailed Mann-Whitney U Test. In (b-c) significances were determined using a 2-tailed Mann-Whitney U Test.

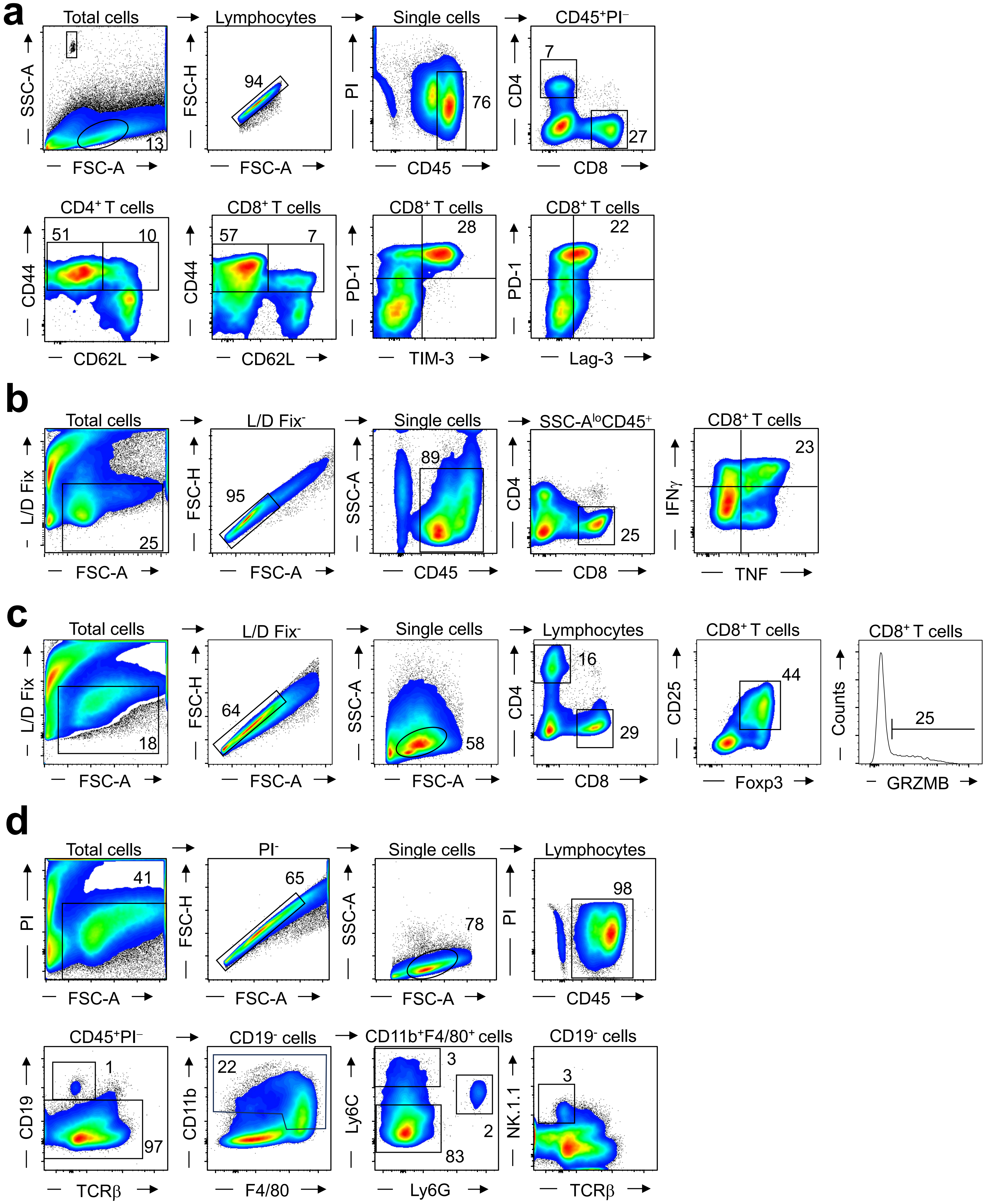
a
Total cells
Lymphocytes
Single cells
CD45+PI−
SSC-A
FSC-A
FSC-H
PI
CD45
CD4
CD8
13
94
76
7
27
CD4+ T cells
CD8+ T cells
CD44
CD62L
PD-1
TIM-3
Lag-3
51
10
57
7
28
22
b
L/D Fix-
SSC-AloCD45+
L/D Fix
IFNγ
TNF
25
95
89
25
23
c
64
58
16
29
44
25
CD25
Foxp3
Counts
GRZMB
18
d
PI-
41
65
78
98
CD19- cells
CD11b+F4/80+ cells
CD19
TCRβ
CD11b
F4/80
Ly6C
Ly6G
NK.1.1
1
97
22
3
2
83
3

Supplementary Fig. 9. Gating strategy for intratumoral lymphocytes. AT3-OVA mammary tumor cells were injected into the fourth inguinal mammary fat pads of C57BL/6 mice. Mice were treated with Compound 182 (10 mg/kg i.v.) or saline on days (d) 6, 8, 10, 12, 14, 16, 18 and 21 after tumor cell implantation. **a**) Tumor-infiltrating lymphocytes were stained with fluorochrome-conjugated antibodies for CD4, CD8, CD44, CD45, CD62L, PD-1, Tim-3 and Lag-3. Live PI^- (propidium iodide) $CD45^+$ single cells were gated for $CD4^+$ and $CD8^+$ T cells and the proportions of $CD4^+$ or $CD8^+CD44^{hi}CD62L^{hi}$ central/memory T cells and $CD4^+$ or $CD8^+CD44^{hi}CD62L^{lo}$ effector/memory T cells and exhausted $CD8^+PD\text{-}1^{hi}TIM\text{-}3^{hi}$ or $CD8^+PD\text{-}1^{hi}Lag\text{-}3^{hi}$ T cells were determined by flow cytometry. **b**) Tumor-infiltrating T cells were stimulated with PMA/Ionomycin in the presence of Golgi Stop/Plug and stained for CD4, CD8, CD45 and intracellular IFN-γ and TNF. Live L/D Fix^- (LIVE/DEAD® Fixable Near IR-stain) $CD45^+$ single cells were gated for $CD8^+$ T cells and the proportions of activated $CD8^+IFN\text{-}\gamma^+TNF^+$ TILs were determined by flow cytometry. **c**) Tumor-infiltrating lymphocytes were stained with fluorochrome-conjugated antibodies for CD4, CD8, CD25 and intracellular FoxP3 and GRZMB. Live L/D Fix^- (LIVE/DEAD® Fixable Near IR-stain) $CD45^+$ single cells were gated for $CD4^+$ and $CD8^+$ T cells and the proportions of $CD4^+CD25^{hi}FoxP3^+$ T_{regs} and $CD8^+GRZMB^+$ cytotoxic T cell were determined by flow cytometry. **d**) Tumor-infiltrating lymphocytes were stained with fluorochrome-conjugated antibodies for CD45, CD19, CD11b, F4/80, Ly6C, Ly6G, NK1.1 and TCRβ. Live PI^- (propidium iodide) $CD45^+$ single cells were gated for $CD19^+$ and $CD19^-$ cells and proportions of $CD19^+$ B cells were determined by flow cytometry. $CD19^-$ cells were gated for $CD11b^{hi/lo}$ and $F4/80^{hi/lo}$ cells and the proportions of $CD11b^{hi/lo}$ $F4/80^{hi/lo}$ monocytic ($Ly6G^{lo}/Ly6C^{hi}$) and granulocytic ($Ly6G^{hi}/Ly6C^{hi}$) myeloid derived suppressor cells (MDSCs) and were determined by flow cytometry. In (a, d) cells were quantified with Flow-Count Fluorospheres® (Beads).

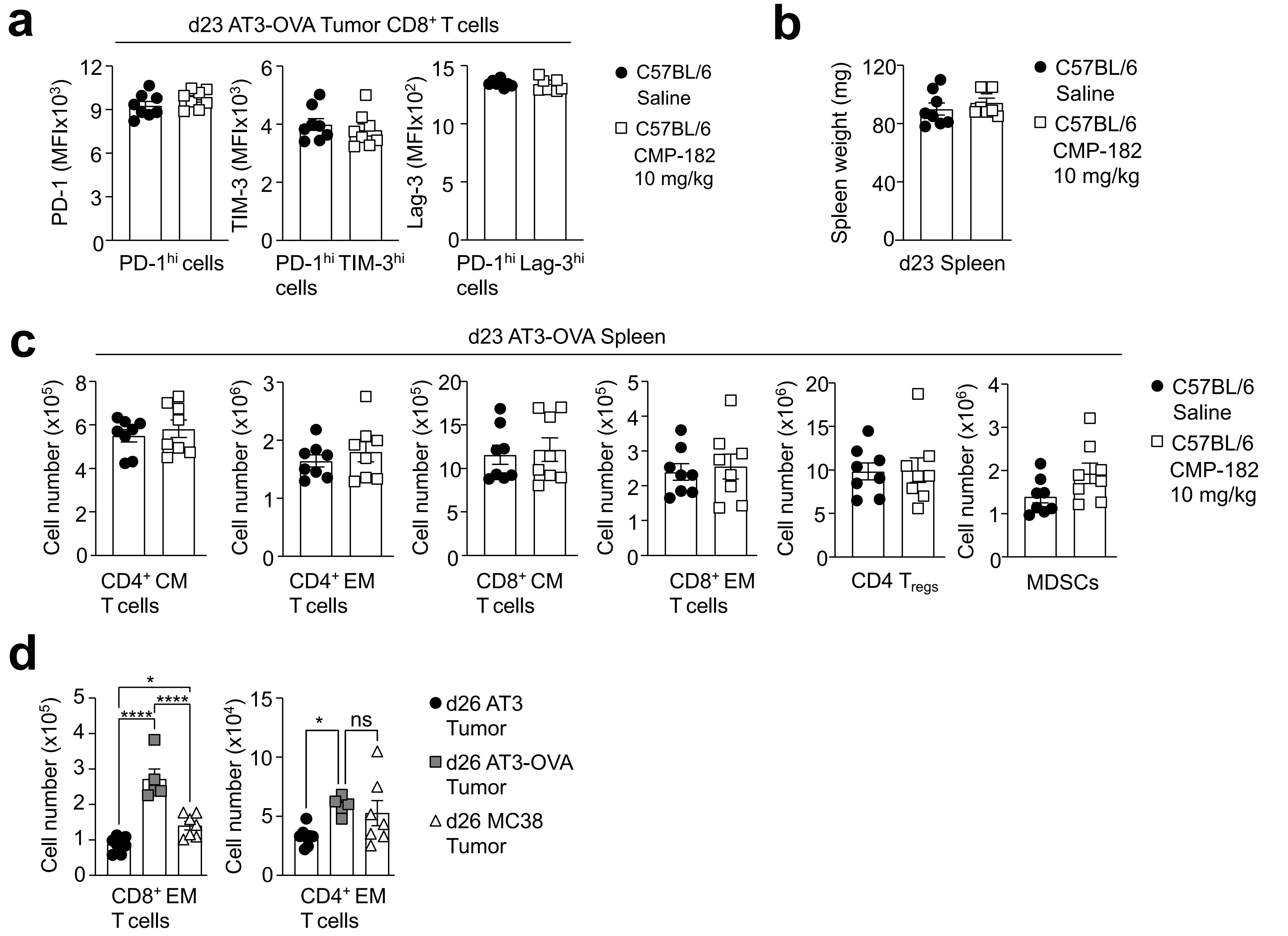
a
d23 AT3-OVA Tumor CD8+ T cells
PD-1 (MFIx10³)
PD-1hi cells
TIM-3 (MFIx10³)
PD-1hi TIM-3hi cells
Lag-3 (MFIx10²)
PD-1hi Lag-3hi cells
C57BL/6 Saline
C57BL/6 CMP-182 10 mg/kg
b
Spleen weight (mg)
d23 Spleen
C57BL/6 Saline
C57BL/6 CMP-182 10 mg/kg
c
d23 AT3-OVA Spleen
Cell number (x10⁵)
CD4+ CM T cells
Cell number (x10⁶)
CD4+ EM T cells
Cell number (x10⁵)
CD8+ CM T cells
Cell number (x10⁵)
CD8+ EM T cells
Cell number (x10⁶)
CD4 Tregs
Cell number (x10⁶)
MDSCs
C57BL/6 Saline
C57BL/6 CMP-182 10 mg/kg
d
Cell number (x10⁵)
CD8+ EM T cells
*
****
****
Cell number (x10⁴)
CD4+ EM T cells
*
ns
d26 AT3 Tumor
d26 AT3-OVA Tumor
d26 MC38 Tumor

Supplementary Fig. 10. Compound 182 does not affect splenic T cell numbers. **a-c**) AT3-OVA mammary tumor cells were injected into the fourth inguinal mammary fat pads of 8 week-old C57BL/6 female mice. Mice were treated with Compound 182 (CMP-182; 10 mg/kg i.v.; n=8) or saline (n=8) on days (d) 6, 8, 10, 12, 14, 16, 18 and 21 after tumor cell implantation. **a**) PD-1, TIM-3 and Lag-3 mean fluorescence intensities (MFI) on PD-1, TIM-3 and Lag-3 on PD-1^{hi} or PD-1^{hi}TIM-3^{hi} or PD-1^{hi}Lag-3^{hi} tumor-infiltrating $CD8^{+}$ T cells were analysed by flow cytometry. **b**) Spleen weights from mice in (a) were determined. **c**) Splenocytes from mice in (a) including $CD44^{hi}CD62L^{hi}$ $CD8^{+}$ and $CD4^{+}$ central/memory (CM) T cells and $CD44^{hi}CD62L^{lo}$ $CD8^{+}$ and $CD4^{+}$ effector/memory (EM) T cells, $CD4^{+}CD25^{+}FoxP3^{+}$ regulatory T cells (T_{regs}) and granulocytic and monocytic $CD11b^{+}F4/80^{hi/lo}Ly6C^{+}Ly6G^{+/-}$ (MDSCs) myeloid-derived suppressor cells were analysed by flow cytometry. **d**) AT3 (n=8) or AT3-OVA (n=5) mammary tumor cells were injected into the fourth inguinal mammary fat pads of 8 week-old C57BL/6 female mice or MC38 (n=7) colorectal tumor cells were xenografted into the flanks of 8 week-old C57BL/6 male mice. $CD8^{+}$ and $CD4^{+}$ EM T cells were analysed by flow cytometry. In (a-d) representative results (means ± SEM) from at least two independent experiments are shown. In (d) significances were determined using a 1-way ANOVA Test.

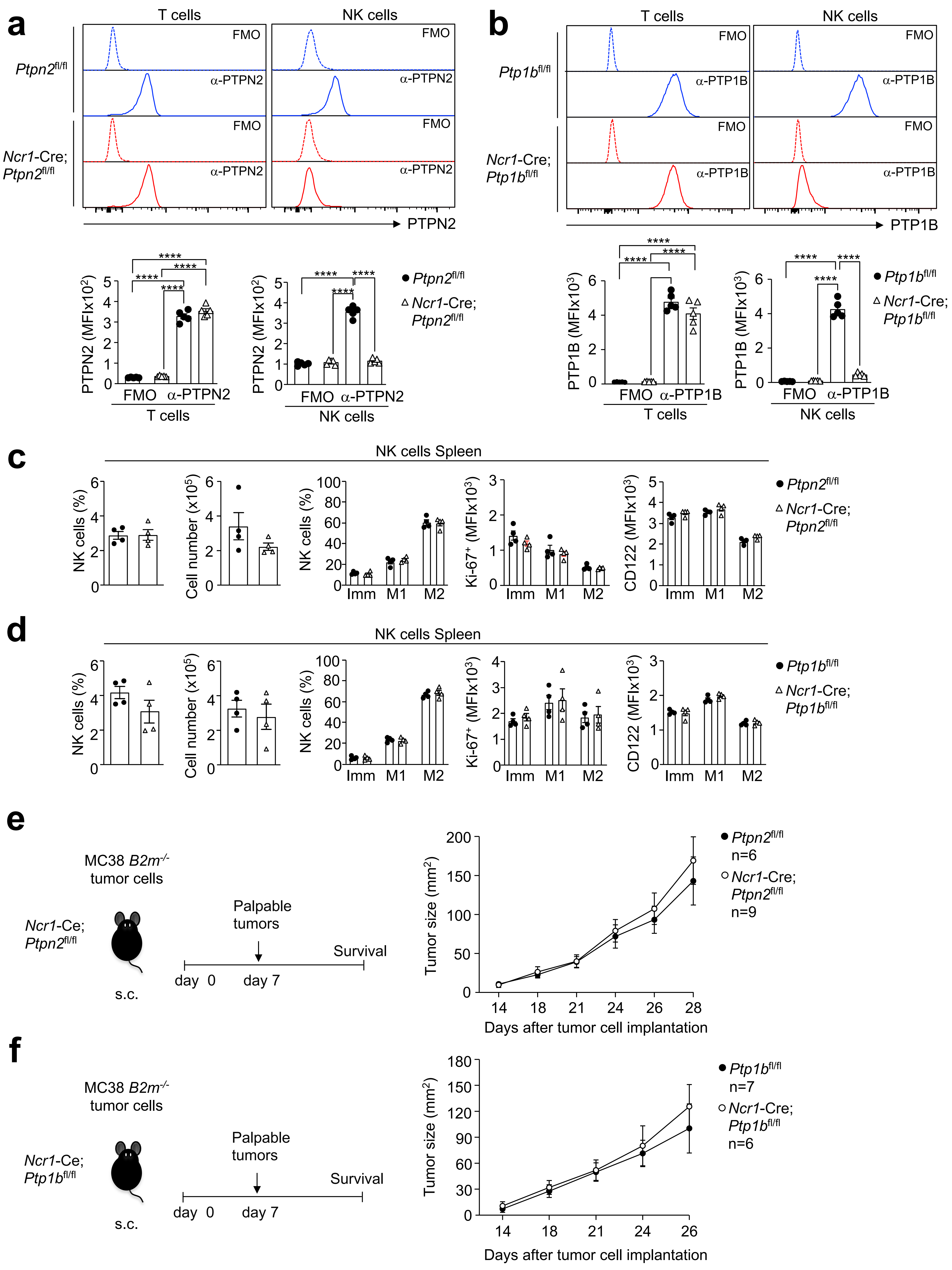
a
T cells
NK cells
Ptpn2fl/fl
Ncr1-Cre; Ptpn2fl/fl
FMO
α-PTPN2
PTPN2
PTPN2 (MFIx10²)
FMO α-PTPN2
T cells
NK cells
****
Ptpn2fl/fl
Ncr1-Cre; Ptpn2fl/fl
b
T cells
NK cells
Ptp1bfl/fl
Ncr1-Cre; Ptp1bfl/fl
FMO
α-PTP1B
PTP1B
PTP1B (MFIx10³)
FMO α-PTP1B
Ptp1bfl/fl
Ncr1-Cre; Ptp1bfl/fl
c
NK cells Spleen
NK cells (%)
Cell number (x10⁵)
Ki-67⁺ (MFIx10³)
CD122 (MFIx10³)
Imm M1 M2
Ptpn2fl/fl
Ncr1-Cre; Ptpn2fl/fl
d
NK cells Spleen
NK cells (%)
Cell number (x10⁵)
Ki-67⁺ (MFIx10³)
CD122 (MFIx10³)
Imm M1 M2
Ptp1bfl/fl
Ncr1-Cre; Ptp1bfl/fl
e
MC38 B2m-/- tumor cells
Ncr1-Ce; Ptpn2fl/fl
s.c.
Palpable tumors
Survival
day 0
day 7
Tumor size (mm²)
Days after tumor cell implantation
Ptpn2fl/fl n=6
Ncr1-Cre; Ptpn2fl/fl n=9
f
MC38 B2m-/- tumor cells
Ncr1-Ce; Ptp1bfl/fl
s.c.
Palpable tumors
Survival
day 0
day 7
Tumor size (mm²)
Days after tumor cell implantation
Ptp1bfl/fl n=7
Ncr1-Cre; Ptp1bfl/fl n=6

Supplementary Fig. 11. NK cell-specific deletion of PTP1B or PTPN2 does not alter NK cell development and tumor growth. **a-b**) Splenic $CD3^+CD19^-CD49b^-NK.1.1^-$ T cells and $CD3^-CD19^-CD49b^{hi}NK.1.1^{hi}$ NK cells from **a**) *Ptpn2*$^{fl/fl}$ (n=5) and *Ncr1*-Cre;*Ptpn2*$^{fl/fl}$ (n=5) or **b**) *Ptp1b*$^{fl/fl}$ (n=5) and *Ncr1*-Cre;*Ptp1b*$^{fl/fl}$ (n=5) C57BL/6 male mice were assessed for PTPN2 and PTP1B protein levels by flow cytometry; FMO staining is background staining. **c-d**) Proportions of NK cells ($CD45^+NK1.1^+$, $CD49b^+NKp46^+CD3^-TCR\beta^-CD19^-F4/80^-$) from **c**) *Ptpn2*$^{fl/fl}$ (n=4) and *Ncr1*-Cre;*Ptpn2*$^{fl/fl}$ (n=4) or **d**) *Ptp1b*$^{fl/fl}$ (n=4) and *Ncr1*-Cre;*Ptp1b*$^{fl/fl}$ C57BL/6 male mice in each maturation subset [immature (Imm. $CD27^+$, $CD11b^-$), M1 ($CD27^+$, $CD11b^+$), M2 ($CD27^-$, $CD11b^+$)] and those staining for Ki67 (a marker of proliferation) or CD122 (IL2Rβ/IL15Rβ) were determined by flow cytometry. **e-f**) MC38 *B2m*$^{-/-}$ tumor cells were xenografted (subcutaneous; s.c) into the flanks of **e**) 8 week-old *Ptpn2*$^{fl/fl}$ (n=6) and *Ncr1*-Cre;*Ptpn2*$^{fl/fl}$ (n=9) C57BL/6 male mice, or **f**) 8 week-old *Ptp1b*$^{fl/fl}$ (n=7) and *Ncr1*-Cre;*Ptp1b*$^{fl/fl}$ (n=6) C57BL/6 male mice and tumor growth was monitored. In (a-f) representative results (means ± SEM) from at least two independent experiments are shown. In (a) significances were determined using a 1-way ANOVA Test.

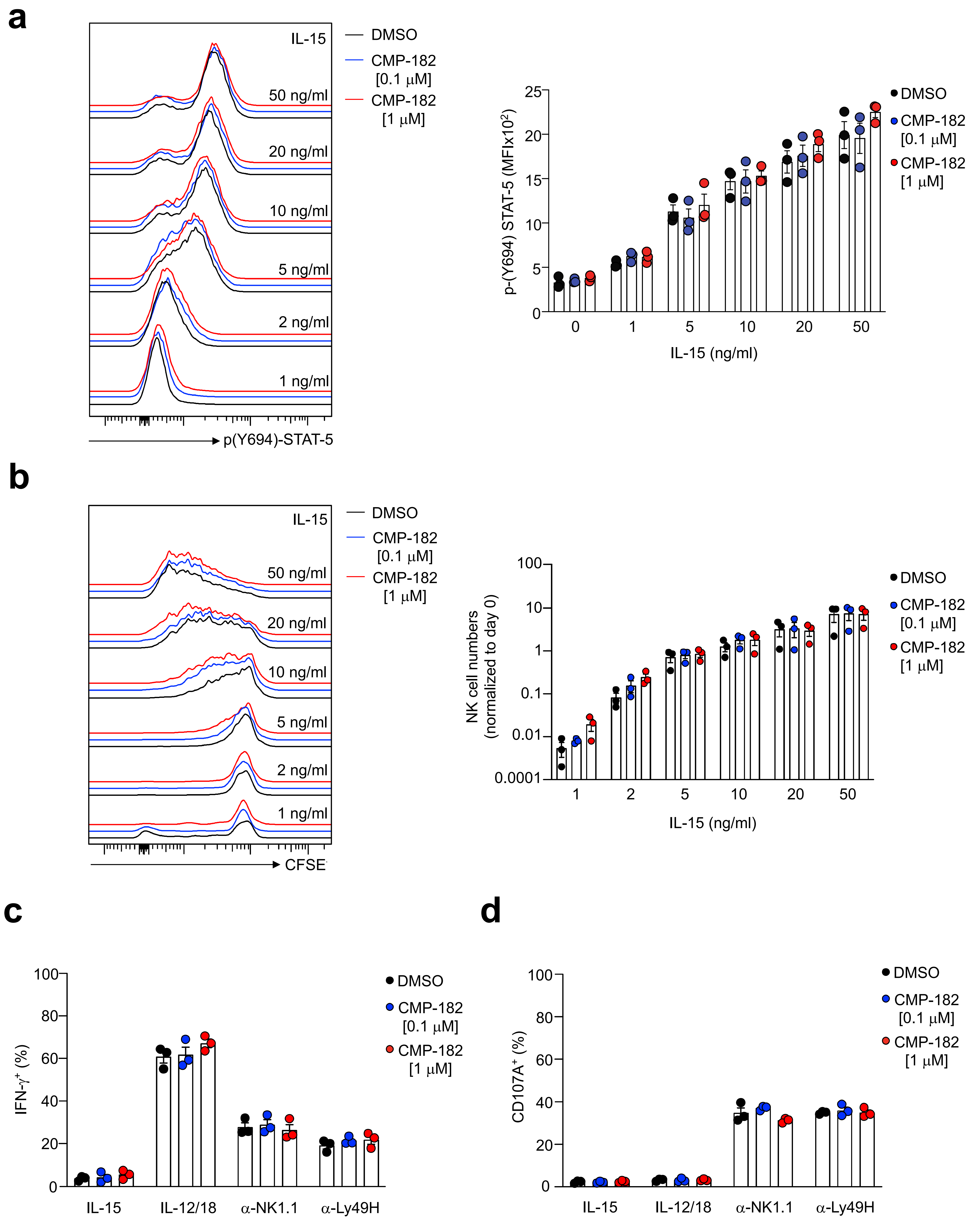

a
IL-15
50 ng/ml
20 ng/ml
10 ng/ml
5 ng/ml
2 ng/ml
1 ng/ml
p(Y694)-STAT-5
DMSO
CMP-182 [0.1 μM]
CMP-182 [1 μM]
p-(Y694) STAT-5 (MFIx10²)
IL-15 (ng/ml)
b
IL-15
CFSE
NK cell numbers (normalized to day 0)
IL-15 (ng/ml)
c
IFN-γ⁺ (%)
IL-15
IL-12/18
α-NK1.1
α-Ly49H
d
CD107A⁺ (%)

Supplementary Fig. 12. Compound 182 does not enhance NK cell cytotoxicity and proliferation in vitro. **a-b**) NK cells from C57BL/6 male mice (n=3 per group) were expanded *ex vivo* in the presence of IL-15 and then pre-treated with Compound 182 (CMP-182; 0.1 or 1 μg/ml) or vehicle (1% v/v DMSO) in the absence of IL-15. **a**) NK cells were re-stimulated for 15 min with the indicated amounts of IL-15 and Y694 phosphorylated STAT-5 (p-STAT-5) mean fluorescence intensities (MFIs) determined by flow cytometry. **b**) Alternatively, NK cells were stained with carboxyfluorescein succinimidyl ester (CFSE, Invitrogen) and then re-stimulated with the indicated amounts of IL-15 for 96 h and NK cell proliferation (CFSE dilution) monitored by flow cytometry. **c-d**) NK cells from C57BL/6 male mice (n=3 per group) were expanded *ex vivo* in the presence of IL-15 and then pre-treated with CMP-182 (0.1 or 1 μg/ml) or vehicle in the absence of IL-15. NK cells were re-stimulated with either IL-12 (10 ng/ml) or IL-18 (100 ng/ml) or with α-NK1.1 or α-Ly49H for 4 h. NK cells were maintained in 10 ng/ml IL-15 throughout the 4 h incubation time. Percentages of **c**) IFN-γ^+ and **d**) CD107A$^+$ NK cells were determined by flow cytometry. In (a-d) results shown are means ± SEM and are representative of at least two independent experiments.

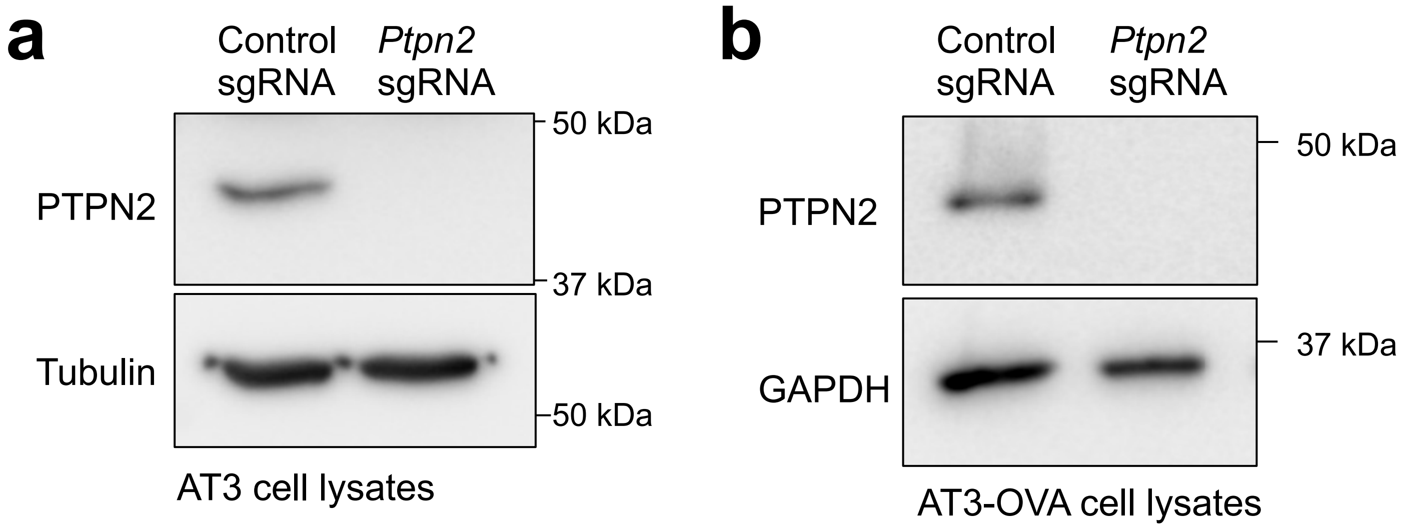
a
Control sgRNA
Ptpn2 sgRNA
PTPN2
Tubulin
50 kDa
37 kDa
50 kDa
AT3 cell lysates
b
Control sgRNA
Ptpn2 sgRNA
PTPN2
GAPDH
50 kDa
37 kDa
AT3-OVA cell lysates

Supplementary Fig. 13. CRISPR/Cas9-mediated PTPN2 deletion in AT3 and AT3-OVA tumor cells. PTPN2 was deleted in **a**) AT3 or **b**) AT3-OVA mammary tumor cells using control or *Ptpn2* sgRNAs and CRISPR RNP gene-editing. Cells were lysed and proteins resolved by SDS-PAGE and immunoblotted for PTPN2 (6F3) and re-probed for either tubulin or actin. Representative results from at least two independent experiments are shown.

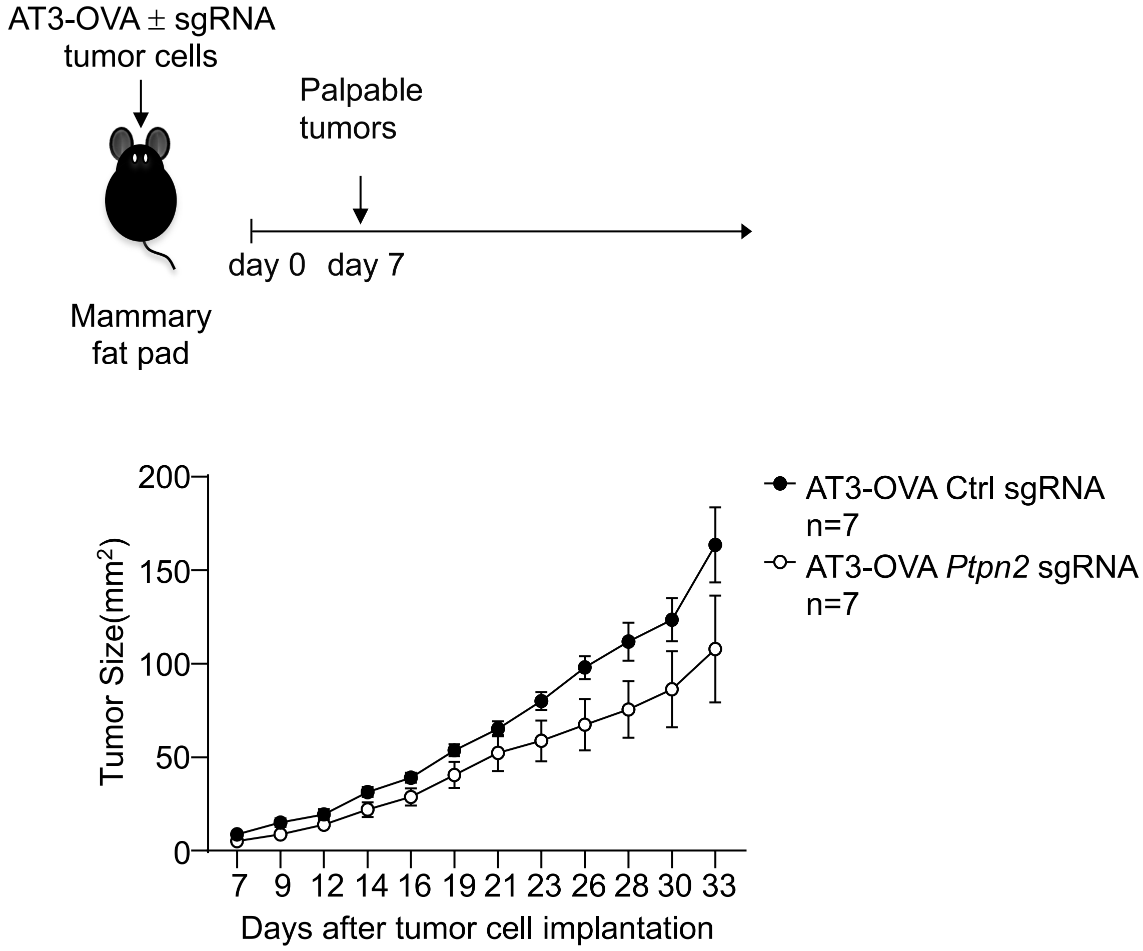

AT3-OVA ± sgRNA
tumor cells
Palpable
tumors
day 0
day 7
Mammary
fat pad
Tumor Size(mm²)
200
150
100
50
0
7
9
12
14
16
19
21
23
26
28
30
33
Days after tumor cell implantation
AT3-OVA Ctrl sgRNA
n=7
AT3-OVA Ptpn2 sgRNA
n=7

Supplementary Fig. 14. PTPN2 deletion does not repress the growth of AT3-OVA mammary tumors. AT3-OVA control cells (Ctl sgRNA; n=7) or those in which PTPN2 had been deleted by CRISPR RNP (*Ptpn2* sgRNA; n=7) were injected into the fourth inguinal mammary fat pads of 8 week-old female C57BL/6 mice and tumor growth was monitored. Representative results (means ± SEM) from at least two independent experiments are shown.

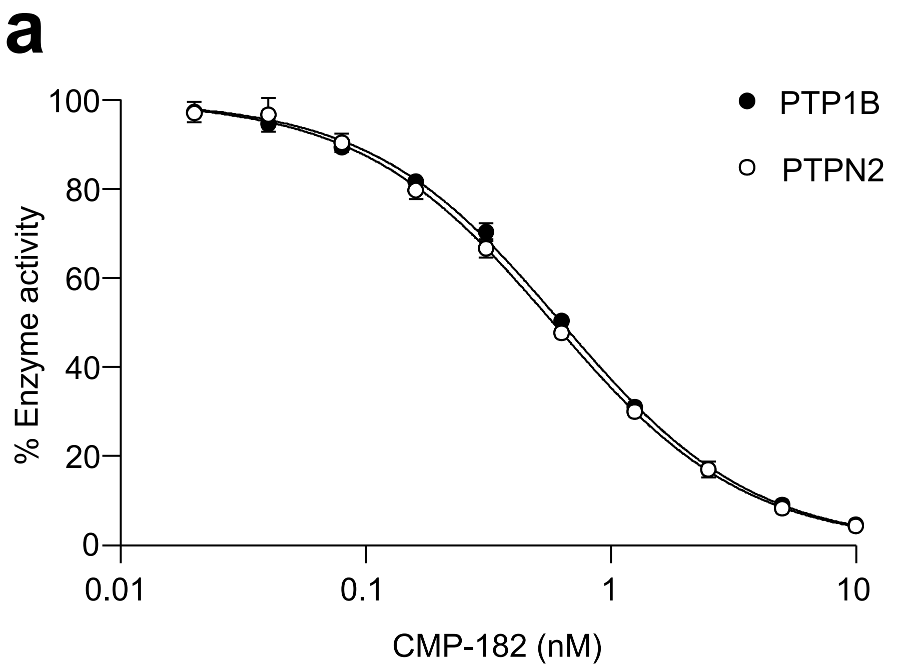
a
PTP1B
PTPN2
% Enzyme activity
100
80
60
40
20
0
0.01
0.1
1
10
CMP-182 (nM)

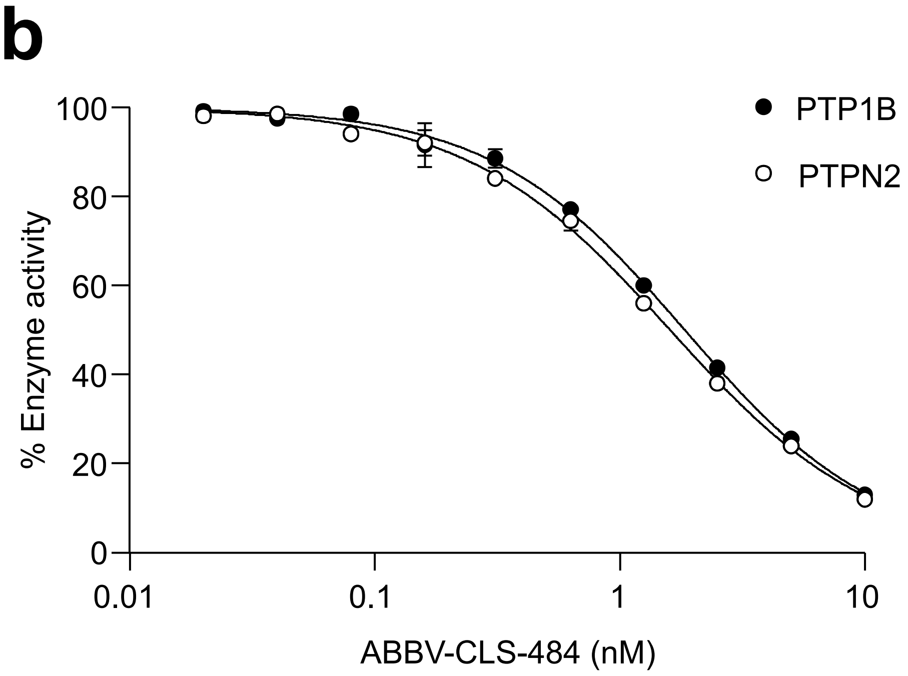
b
PTP1B
PTPN2
% Enzyme activity
100
80
60
40
20
0
0.01
0.1
1
10
ABBV-CLS-484 (nM)

Supplementary Fig. 15. Inhibition of PTP1B and PTPN2 by Compound 182 and ABBV-CLS-484. Effects of **a**) Compound 182 (CMP-182) versus **b**) ABBV-CLS-484 on PTP1B- and PTPN2-catalysed DiFMUP (10 μM) hydrolysis. Results shown are means ± SD and are representative of two independent experiments.
