# Supplementary material for: A small molecule inhibitor of PTP1B and PTPN2 enhances T cell anti-tumor immunity
Source: Nat Commun. 2023 Jul 27;14:4524. doi: 10.1038/s41467-023-40170-8 (PMC10374545; doi:10.1038/s41467-023-40170-8)
Supplement: Supplementary file 3 — Reporting Summary [file 41467_2023_40170_MOESM3_ESM.pdf]

nature portfolio

Corresponding author(s): Tony Tiganis
Florian Wiede

Last updated by author(s): Jul 9, 2023

Reporting Summary

Nature Portfolio wishes to improve the reproducibility of the work that we publish. This form provides structure for consistency and transparency in reporting. For further information on Nature Portfolio policies, see our Editorial Policies and the Editorial Policy Checklist.

Statistics

For all statistical analyses, confirm that the following items are present in the figure legend, table legend, main text, or Methods section.

n/a	Confirmed	
☐	☒	The exact sample size (*n*) for each experimental group/condition, given as a discrete number and unit of measurement
☐	☒	A statement on whether measurements were taken from distinct samples or whether the same sample was measured repeatedly
☐	☒	The statistical test(s) used AND whether they are one- or two-sided<br>*Only common tests should be described solely by name; describe more complex techniques in the Methods section.*
☒	☐	A description of all covariates tested
☐	☒	A description of any assumptions or corrections, such as tests of normality and adjustment for multiple comparisons
☐	☒	A full description of the statistical parameters including central tendency (e.g. means) or other basic estimates (e.g. regression coefficient) AND variation (e.g. standard deviation) or associated estimates of uncertainty (e.g. confidence intervals)
☐	☒	For null hypothesis testing, the test statistic (e.g. *F*, *t*, *r*) with confidence intervals, effect sizes, degrees of freedom and *P* value noted<br>*Give P values as exact values whenever suitable.*
☒	☐	For Bayesian analysis, information on the choice of priors and Markov chain Monte Carlo settings
☒	☐	For hierarchical and complex designs, identification of the appropriate level for tests and full reporting of outcomes
☒	☐	Estimates of effect sizes (e.g. Cohen's *d*, Pearson's *r*), indicating how they were calculated

Our web collection on statistics for biologists contains articles on many of the points above.

Software and code

Policy information about availability of computer code

Data collection	BD FACS DIVA Software v8.0.1; Slides for histology & immunohistochemistry were scanned using an Aperio Scanscope AT Turbo
Data analysis	Graphpad Prism 9.5.1; FlowJo v10.8.2; Images for histology & immunohistochemistry were analysed using OlyVIA imaging software; PHENIX with Coot

For manuscripts utilizing custom algorithms or software that are central to the research but not yet described in published literature, software must be made available to editors and reviewers. We strongly encourage code deposition in a community repository (e.g. GitHub). See the Nature Portfolio guidelines for submitting code & software for further information.

Data

Policy information about availability of data

All manuscripts must include a data availability statement. This statement should provide the following information, where applicable:

- Accession codes, unique identifiers, or web links for publicly available datasets
- A description of any restrictions on data availability
- For clinical datasets or third party data, please ensure that the statement adheres to our policy

The source data underlying Figures 2, 3, 4, 5, 6a-d, f, 7a-d, 9a-f, 10a, c, e-g and Supplementary Figures 6-13 are provided as a Source Data file. Crystal structure atomic coordinates have been deposited in the Protein Data Bank under accession code 8SKL (https://doi.org/10.2210/pdb8skl/pdb). Other raw datasets generated during this study and all published non-commercial reagents can be made available from the corresponding author on reasonable request.

Research involving human participants, their data, or biological material

Policy information about studies with human participants or human data. See also policy information about sex, gender (identity/presentation), and sexual orientation and race, ethnicity and racism.

Reporting on sex and gender	N/A
Reporting on race, ethnicity, or other socially relevant groupings	N/A
Population characteristics	N/A
Recruitment	N/A
Ethics oversight	N/A

Note that full information on the approval of the study protocol must also be provided in the manuscript.

Field-specific reporting

Please select the one below that is the best fit for your research. If you are not sure, read the appropriate sections before making your selection.

☒ Life sciences ☐ Behavioural & social sciences ☐ Ecological, evolutionary & environmental sciences

For a reference copy of the document with all sections, see nature.com/documents/nr-reporting-summary-flat.pdf

Life sciences study design

All studies must disclose on these points even when the disclosure is negative.

Sample size	No statistical methods were used to predefine the sample size for each experiments but sample sizes are similar to those in our previous publications [(Wiede, F. et al. PTP1B Is an Intracellular Checkpoint that Limits T-cell and CAR T-cell Antitumor Immunity. Cancer Discov 12, 752-773, (2022); Wiede, F. et al PTPN2 phosphatase deletion in T cells promotes anti-tumour immunity and CAR T-cell efficacy in solid tumours. EMBO J 39, e103637, (2020)].<br>For in vivo studies the final sample size was dependent on several factors (e.g. mice bearing undersized tumors or subcutaneous growing mammary tumours) which dictated the number of tumor-bearing experimental mice.<br>For in vitro studies sample size was determined through our previous experience where we have aimed for samples sizes at least > 4. This ensured the correct application of a non-parametric Mann Whitney U test.
Data exclusions	No specific data exclusions were undertaken in this manuscript with the exception of mice where mammary AT3/AT3-OVA and subcutaneous MC38 tumors failed to establish, were too small or were found to be growing subcutaneously. These mice were excluded from FACS, qRT-PCR and histological analysis.
Replication	Each experiment was performed and successfully repeated at least two times unless otherwise stated in the figure legends.
Randomization	Tumor-bearing mice were not randomized but rather evenly distributed according to tumor sizes between the groups. This ensured equal tumor sizes between the different groups at the start of the experiment.
Blinding	Blinding was generally not performed for this study with the exception of experiments shown in Fig. 6a and Fig. S13. Here tumor measurements were performed blinded by de-identifying animals.

Reporting for specific materials, systems and methods

We require information from authors about some types of materials, experimental systems and methods used in many studies. Here, indicate whether each material, system or method listed is relevant to your study. If you are not sure if a list item applies to your research, read the appropriate section before selecting a response.

Materials & experimental systems

n/a	Involved in the study
☐	☒ Antibodies
☐	☒ Eukaryotic cell lines
☒	☐ Palaeontology and archaeology
☐	☒ Animals and other organisms
☒	☐ Clinical data
☒	☐ Dual use research of concern
☒	☐ Plants

Methods

n/a	Involved in the study
☒	☐ ChIP-seq
☐	☒ Flow cytometry
☒	☐ MRI-based neuroimaging

Antibodies

Antibodies used

Flow cytometry:
Phycoerythrin (PE) or peridinin chlorophyll protein-cyanine 5.5 (PerCP-Cy5.5)-conjugated CD3 (BD Biosciences Cat#561808 [1-100], BD Biosciences Cat# 55163 [1-100]); Pacific Blue-conjugated (PB), PerCP-Cy5.5 or phycoerythrin-cyanine 7 (PE-Cy7)-conjugated CD4 (BD Biosciences Cat# 558170 [1-200]; BD Biosciences Cat# 550954 [1-400], BD Biosciences Cat# 561099 [1-1000]); Brilliant Violet (BV) 711, Allophycocyanin (APC), PB or Brilliant Ultra Violet (BUV) 395-conjugated CD8 (BioLegend Cat# 100759 [1-400]; BD Biosciences Cat# 558106 [1-400]; BD Biosciences Cat#565968 [1-500]); BUV805 or BV711-conjugated CD11b (BD Biosciences Cat# 741934 [1-400]; BioLegend Cat# 101241 [1-400]); APC-conjugated CD11c (BioLegend Cat# 117309 [1-400]); PE-Cy5 or PE-Cy7-conjugated CD19 (Biolegend Cat# 115510 [1-400], BD Biosciences Cat# 552854 [1-400]); PE-conjugated CD25 (BD Biosciences Cat# 553866 [1-600]); BV650-conjugated CD27 (BD Biosciences Cat# 740491 [1-800]); Fluorescein isothiocyanate (FITC) or BV786-conjugated CD44 (BD Biosciences Cat# 553133 [1-1000]; BD Biosciences Cat#563736 [1-500]); APC or APC-eFluor780-conjugated CD45 (Thermo Fisher Scientific Cat# 17-0451-83 [1-500], Thermo Fisher Scientific Cat# 47-0451-82 [1-500]); BUV563-conjugated CD49b (BD Biosciences Cat# 741280 [1-200]); BV480-conjugatd Ly-49H (BD Biosciences Cat# 746493 [1-200]); APC, BV421, PE or BUV737-conjugated CD62L (BD Biosciences Cat# 553152 [1-1000]; BioLegend Cat# 104435 [1-500]; BD Biosciences Cat# 553151 [1-1000]; BD Biosciences Cat# 612833 [1-500]); PE-conjugated CD107a (BD Bioscience Cat# 558661 [1-200]); FITC or PE-Cy7-conjugated CD279 (BioLegend Cat# 135213 [1-400]; BioLegend Cat# 135216 [1-400]); PE-Cy7-conjugated CD335 (Biolegend Cat# 137618 [1-400]); PE-Cy7-conjugated CD366 (BioLegend Cat# 134010 [1-400]); BV605 or PerCP-Cy5.5-conjugated TCR-beta (BioLegend Cat# 109241 [1-500]; BD Biosciences Cat# 560657 [1-200]); BV421-conjugated NK1.1 (PK136; BD Biosciences Cat# 562921 [1-200]); APC-Cy7-conjugated Ly6C (BD Biosciences Cat# 560596 [1-800]); FITC-conjugated Ly6G (BD Biosciences Cat# 561105 [1-800]); PE or PE-Cy5-conjugated F4/80 (BD Biosciences Cat# 565410 [1-200], Biolegend Cat# 123112 [1-200]); PE-Cy7-conjugated IFN-gamma (BD Biosciences Cat# 557649 [1-400], Thermo Fisher Scientific Cat# 25-7311-82 [1-400]); APC-conjugated TNF (BD Biosciences Cat# 561062 [1-200]); Alexa Fluor 647 or BV421-conjugated Granzyme B (BioLegend Cat# 515405 [1-400], BD Biosciences Cat#563389 [1-400]); FITC-conjugated Foxp3 (Thermo Fisher Scientific Cat# 11577382 [1-200]); eFlor660-conjugated phospho-SRC (Tyr418) (Invitrogen Cat# 50-9034-42 [1-200]).

Others:
CD3 (BD Biosciences Cat# 553058) and CD28 (BD Biosciences Cat# 557393) antibodies for T cell stimulation and NK1.1 (BD Biosciences Cat# 564143) antibody for NK cell stimulation were purchased from BD Biosciences. Ly49H (clone 3D10, BioLegend Cat# 144702) antibody for NK stimulation was from BioLegend. Antibody concentrations for T cell and NK stimulations are described in the methods and in each corresponding figure legend.
For analysing T cell signaling, p-(Y418) SFK (Thermo Fisher Scientific Cat# 14-9034-82 [1-1000]) was from Invitrogen and p-(Y694) STAT-5 (D47E7) XP® (Cell Signaling Technology Cat# 4322 [1-400]) was from Cell Signaling.
InVivoMAb anti-mouse PD-1 (Bio X Cell Cat# BE0146) and InVivoMAb rat IgG2a isotype control (Bio X Cell Cat# BE0251) were purchased from Bio X Cell.
Rabbit monoclonal PTP1B (Abcam Cat# ab244207 [1-200]) antibody for flow cytometry was from Abcam and mouse monoclonal PTPN2 (clone: 6F3) antibody [1-100] for immunoblotting and flow cytometry was from MediMabs. Mouse anti-actin (Thermo Fisher Scientific Cat# MA5-11866 [1-2000]) from Invitrogen and mouse α-tubulin (Sigma-Aldrich Cat# T5168 [1-4000]) was from Merck.
PTP1B monoclonal rabbit antibody and p-STAT-5 were detected by the secondary antibody against rabbit IgG (H+L) F(ab')2 fragment conjugated to Alexa Fluor 647 (Jackson ImmunoResearch Labs Cat# 111-606-144 [1-800]).
PTPN2 monoclonal mouse antibody was detected by the secondary antibody against a mouse IgG (H+L) F(ab')2 fragment conjugated to AlexaFluor 647 (Molecular Probes/Thermo Fisher Scientific Cat# A-21237 [1-800]).

Validation

All antibodies are routinely validated by manufacturer and validations are available on the manufacturer's websites. Antibodies for flow cytometry were titrated on mouse lymphocytes prior to usage. PTP1B monoclonal rabbit antibody and PTPN2 monoclonal mouse antibody were validated on PTP1B null and PTPN2 null T and NK cells.

Eukaryotic cell lines

Policy information about cell lines and Sex and Gender in Research

Cell line source(s)

All cell lines used in this study were originally purchased from American Type Culture Collection (ATCC). The C57BL/6 murine breast tumor cell line AT-3 was genetically engineered to express chicken ovalbumin and have been previously described: AT3/AT3-OVA [Loi et al., CD73 promotes anthracycline resistance and poor prognosis in triple negative breast cancer. Proc Natl Acad Sci U S A 110, 11091-11096 (2013)]. The C57BL/6 murine colon carcinoma cell line MC38 was described in [A. Redeker et al., The Quantity of Autocrine IL-2 Governs the Expansion Potential of CD8+ T Cells. J Immunol 195, 4792-4801 (2015)] and the corresponding B2m–/– MC38 cells in [Wolf, N. K. et al. Synergy of a STING agonist and an IL-2 superkine in cancer immunotherapy against MHC I-deficient and MHC I+ tumors. Proc Natl Acad Sci USA 119, e2200568119, (2022)]. PTPN2 was deleted in AT3 or AT3-OVA mammary tumor cells using CRISPR Ribonucleoprotein (RNP) gene-editing as

described previously [Goh, P. K. et al. PTPN2 elicits cell autonomous and non-cell autonomous effects on antitumor immunity in triple-negative breast cancer. Sci Adv 8, eabk3338, (2022)].

Authentication	All cell lines have been previously maintained in-house and were therefore not authenticated.
Mycoplasma contamination	All cell lines used in this study were routinely tested for mycoplasma contamination. No contaminations were detected.
Commonly misidentified lines (See ICLAC register)	No commonly misidentified cell lines were used.

Animals and other research organisms

Policy information about studies involving animals; ARRIVE guidelines recommended for reporting animal research, and Sex and Gender in Research

Laboratory animals	Mice were maintained on a 12 h light-dark cycle in a temperature-controlled (22 degree Celsius and 40-50% humidity) high barrier facility with free access to food and water. 8-12 week old Ptp1bfl/fl, Ptpn2fl/fl, Lck-Cre;Ptp1bfl/fl, Lck-Cre;Ptpn2fl/fl and Mx1-Cre;Rosa26-eYFP;Ptpn2fl/fl (referred to as Mx1-Cre; Ptpn2fl/fl) mice on a C57BL/6 background have been described previously [Wiede, F. et al. PTP1B Is an Intracellular Checkpoint that Limits T-cell and CAR T-cell Antitumor Immunity. Cancer Discov 12, 752-773, (2022); Wiede, F. et al. T cell protein tyrosine phosphatase attenuates T cell signaling to maintain tolerance in mice. J Clin Invest 121, 4758-4774, (2011); Wiede, F., Sacirbegovic, F., Leong, Y. A., Yu, D. & Tiganis, T. PTPN2-deficiency exacerbates T follicular helper cell and B cell responses and promotes the development of autoimmunity. J Autoimmun 76, 85-100, (2017)]. Ncr1-Cre (C57BL/6) mice [Narni-Mancinelli, E. et al. Fate mapping analysis of lymphoid cells expressing the NKp46 cell surface receptor. Proc Natl Acad Sci U S A 108, 18324-18329, (2011)] were crossed with Ptp1bfl/fl (C57BL/6) or Ptpn2fl/fl (C57BL/6) to generate Ncr1-Cre;Ptp1bfl/fl and Ncr1-Cre;Ptpn2fl/fl mice respectively. C57BL/6/J mice were purchased from Monash Animal Research Platform (MARP, Clayton, Australia), the WEHI Animal Facility (Kew, Australia) or from the Animal Resource Centre (ARC, Perth, Australia).
Wild animals	This study did not involve wild animals.
Reporting on sex	Sex-matched littermates were used in all experiments; unless otherwise indicated, female donor mice were used for all in vivo experiments, whereas mice of the same sex were used for ex vivo experiments.
Field-collected samples	This study did not involve field-collected samples.
Ethics oversight	All animal experiments were performed in accordance with the NHMRC Australian Code of Practice for the Care and Use of Animals. All protocols were approved by the Monash University School of Biomedical Sciences Animal Ethics Committee (Ethics numbers: 23177, 36697).

Note that full information on the approval of the study protocol must also be provided in the manuscript.

Flow Cytometry

Plots

Confirm that:

☒ The axis labels state the marker and fluorochrome used (e.g. CD4-FITC).

☒ The axis scales are clearly visible. Include numbers along axes only for bottom left plot of group (a 'group' is an analysis of identical markers).

☒ All plots are contour plots with outliers or pseudocolor plots.

☒ A numerical value for number of cells or percentage (with statistics) is provided.

Methodology

Sample preparation	Single cell suspensions from mouse spleens and lymph nodes were obtained by gently mashing the excised organs through 40 µm cell strainers (BD Biosciences). Cells were incubated for 7 minutes on ice in Red Blood Cell Lysing Buffer Hybri-Max™ (Sigma-Aldrich) for the removal of contaminating erythrocytes and washed with D-PBS/2%FBS and processed for flow cytometry.<br>Tumors were excised and digested at 37°C for 30 min using a cocktail of 1 mg/ml collagenase type IV (Worthington Biochemicals) and 0.02 mg/ml DNase (Sigma-Aldrich) in DMEM supplemented with D-PBS/2%FBS. Cells were passed through a 70 µm cell strainer (BD Biosciences) and processed for flow cytometry.
Instrument	BD LSRII, BD Fortessa X-50, BD Symphony A3
Software	BD FACSDIVA Software v8.0.1; FlowJo v10.8.2
Cell population abundance	Single cell suspensions obtained from freshly isolated lymph nodes of OT-I mice contain generally 50-60% ovalbumine restricted CD8+ T cells, lymphocytes from the lymph nodes of C57BL/6 mice usually contained ~40% CD4+ and ~25-30% CD8+ T cells. The frequency of CD62L+CD44- naive T cells within the CD8+ OT-I T cell population was generally between 90-95%. The frequencies of CD8+ T cells from C57BL/6 mice contained usually 75-80% CD62L+CD44- naive T cells .<br>The relevant cell population for each specific experiment is outlined in each figure.

Gating strategy

All analyses begin with gating for the lymphocyte population (SSC-A versus FSC-A). We next gate on the single-cell population to exclude cell aggregates (FSC-H versus FSC-A). Where possible we have used propedium-iodide or fixable life-dead dyes to exclude dead cells.

☒ Tick this box to confirm that a figure exemplifying the gating strategy is provided in the Supplementary Information.
